# Global Rise of Depression Prevalence Amid the COVID-19 Pandemic

**DOI:** 10.1093/geroni/igab046.1579

**Published:** 2021-12-17

**Authors:** Anna Egbert, Stephen Karpiak, Richard Havlik, Sadiye Cankurtaran, Sirinnaz Ozturk

**Affiliations:** 1 Ronin Institute, Montclair, New Jersey, United States; 2 GMHC, National Resource Center on Aging and HIV, New York, New York, United States; 3 National Institutes of Health, National Institute of Aging, New York, New York, United States; 4 Jagiellonian University, Kraków, Malopolskie, Poland; 5 University of Warsaw, Warsaw, Mazowieckie, Poland

## Abstract

The immense burden of depressive disorders is on the rise, with global prevalence estimates in 2017 ranging from 4% to 13%. The novel coronavirus SARS-CoV-2 is likely to impact the established risk factors for depressive disorders. Thus, a rapid increase in depression prevalence can be expected amid the COVID-19 pandemic. Using epidemiologic data (N=111,225) derived from an extant online survey “Measuring Worldwide COVID-19 Attitudes and Beliefs” (launched by Fetzer and colleagues, March-April 2020) in 178 countries, we examined age-dependent global prevalence of depression and assessed the impact of social factors caused by the COVID-19 pandemic on depressive symptomatology. Point prevalence of depression was measured using the PHQ8 standard cut-off score (i.e., ≥10). Correlates of depressive symptoms were analyzed with hierarchical regression modeling separately in three age groups, i.e., 18-34, 35-54 and 55+ years. We found that nearly 20% of individuals globally revealed significant symptoms of depression, including 27% of young, 15% middle-aged, 9% adults aged 55+. These data suggest that the prevalence of depression is 2-5 times higher than global estimates preceding the COVID-19 pandemic. Regression modeling explained approx. 50% variability in depressive symptoms across the three age groups. Increased risk of depression was found in females, single or divorced individuals, and those who presented poorer health and higher anxiety. Social restrictions amid the COVID-19 pandemic were marginal risks for depression. Together, this study highlights the impact of the COVID-19 pandemic on the mental health of people of different ages and urges the development of increased access to psychological interventions.

